# Developing a Triple Transgenic Cell Line for High-Efficiency Porcine Reproductive and Respiratory Syndrome Virus Infection

**DOI:** 10.1371/journal.pone.0154238

**Published:** 2016-05-16

**Authors:** Linlin Zhang, Zhengzhi Cui, Lei Zhou, Youmin Kang, Li Li, Jinxiu Li, Yunping Dai, Shuyang Yu, Ning Li

**Affiliations:** 1 State Key Laboratory of Agrobiotechnology, College of Biological Sciences, China Agricultural University, Beijing, 100193, China; 2 Key Laboratory of Zoonosis of Ministry of Agriculture, College of Veterinary Medicine, China Agricultural University, Beijing, 100193, China; University of Georgia, UNITED STATES

## Abstract

Porcine reproductive and respiratory syndrome virus (PRRSV) is one of the most devastating pathogens in the swine industry worldwide. Due to the lack of robust cell lines and small animal models, the pathogenesis of PRRSV infection and mechanism for protective vaccination are still not yet well understood. To obtain useful cell lines, several groups have attempted to construct different transgenic cell lines with three PRRSV receptors: CD163, CD169, and CD151. The results showed that CD163 is essential for PRRSV entry into target cells and replication, and both CD169 and CD151 play key roles during PRRSV infection. However, their interplay and combined effect remains unclear. In this study, we generated transgenic BHK-21 derived cell lines co-expressing different combinations of the three receptors, which were transfected with CD163 alone, or the combination of CD163 and CD169, or the combination of CD163 and CD151, or the combination of CD163, CD169, and CD151 using the PiggyBac transposon system. Our results showed that the synergistic interaction among the three receptors was important to improve the susceptibility of cells during PRRSV infection. Through a series of comparable analyses, we confirmed that the cell line co-expressing triple receptors sustained viral infection and replication, and was superior to the current cell platform used for the PRRSV study, MARC-145 cells. Moreover, we found that PRRSV infection of the transgenic cell lines could trigger IFN-stimulated gene responses similar to those of porcine alveolar macrophages and MARC-145 cells. In summary, we developed a stable transgenic cell line susceptible to PRRSV, which may not only provide a useful tool for virus propagation, vaccine development, and pathogenesis studies, but also establish the foundation for small animal model development.

## Introduction

Porcine reproductive and respiratory syndrome (PRRS) is one of the most serious contagious diseases in pigs and causes significant economic losses in the global swine industry [1,2]. The causative pathogen of the disease is porcine reproductive and respiratory syndrome virus (PRRSV), which is a member of *Nidovirales*, family *Arteriviridae*. PPRSV is an enveloped positive-stranded RNA virus containing at least 10 open reading frames (ORFs) [3,4]. PRRSV infection can cause severe reproductive problems, including premature farrowings, stillborn or mummified piglets in sows, or weak piglets that die soon after birth. In addition to reproductive failure, more complicated clinical signs can be characterized by anorexia, fever, lethargy, pneumonia, respiratory failure, and red/blue discoloration of the ears and vulva depending on the age of pigs, type of PRRSV strain, co-infections, and captive conditions [5,6].

PRRSV shows a strict cell and host tropism restriction. It preferentially infects monocytes and macrophages in swine, but there is no evidence to support its ability to infect other species. Porcine alveolar macrophages (PAMs) are the primary target cells supporting PRRSV productive replication both *in vivo* and *in vitro* [7,8]. However, peripheral monocytes/macrophages (particularly peripheral blood monocytes) are largely refractory to PRRSV infection. PRRSV enters target cells by receptor-mediated endocytosis through clathrin-coated vesicles [9–11]. To date, three dominant receptors on PAMs contributing to PRRSV infection have been identified: heparan sulphate (HS), CD169, and CD163 [12–19]. First, PRRSVs attach to HS on PAMs via the viral M/GP5 complex, a glycoprotein dimer present on the viral envelope [14–16]. Subsequently, the virus binds stably to the N-terminus of sialoadhesin (CD169) and is internalized via a process of clathrin-mediated endocytosis [14,15]. Upon internalization, CD163 interacts with the PRRSV GP2 and GP4 glycoproteins and promotes uncoating and release of viral genome from the early endosome into the cytoplasm [17–19]. Previous studies identified several PRRSV-insensitive cells lines, including BHK-21, PK-15, and NLFK, which became fully susceptible after CD163 overexpression [17,20]. On the contrary, immortalized PAMs (CRL-2843) lacking the CD163 receptor became resistant to PRRSV infection [21], and fully recovered after CD163 was regained [22]. In addition, a recent study demonstrated that pigs with defective CD163 were resistant to PRRSV [23]; however, *CD169* ^*-/-*^ pigs could be infected with PRRSV to the same degree as wild-type pigs [24]. These data demonstrated that CD163 plays a critical role in PRRSV entry and replication [18,25], and CD163 alone allows non-permissive cells to be permissive to PRRSV. In addition, co-expression of CD169 and CD163 promotes efficient PRRSV infection [18,26].

Although there is no evidence to show that PRRSV is aggressive in primates, such as monkeys and humans, African green monkey kidney-derived cell lines can be efficiently infected, including MA-104 and MARC-145 cells [27–29]. Based on previous reports, we know that simian vimentin and CD151 play key roles as receptors during MARC-145 cell infected with PRRSV [30,31]. Vimentin mediates the transport of viral particles to the cytosol by binding with cytoskeletal filaments [30], and CD151 may interact with the 3’ UTR of PRRSV RNA [31]. Recently, Huang et al. identified porcine CD151, which could render PK-15 cells susceptible to PRRSV [32]. To date, the precise roles of these two proteins in PRRSV infection and replication are poorly understood.

PAMs, as the primary target cells for PRRSV infection, remain the most efficient cells for PRRSV infection and propagation *in vitro*, but are rarely used for virus proliferation and vaccine production because of the difficulties obtaining and performing large-scale culture. Currently, PRRSV vaccines are mainly produced by MARC-145 cells. However, since the viruses enter and replicate in MARC-145 cells through different pathways with primary macrophages [30,31], adaptation is required for growth [33]. Thus, mutations could accumulate in critical neutralizing epitopes during passaging, resulting in the loss of antigenicity [34,35]. BHK-21 cell line is a powerful tool, having a wide range of applications in animal virology research including gene delivery, virus infection [17], and vaccine virus strain production [26]. Especially, BHK-21 cells can be induced to grow in suspension, which significantly increases the production of virus and vaccine strains. Moreover, BHK-21 cells grow faster, and are easier to maintain, compared with MARC-145 cells; thus, we modified BHK-21 cells to generate a new type of cell line for PPRSV studies.

The PiggyBac (PB) transposon system, a DNA-based transposable system derived from the cabbage lopper moth, was first identified in insect cells [36]. Since it was shown to be efficient in gene transfer in mice in 2005 [37], PB has been shown to have high cargo capacity (more than 100 kb) and high transposition efficiency [38]. Thus, the PB transposon system is commonly used to mediate genomic modification in mammalian cells, such as constructing transgenic cell lines, transgenic animals [39,40], and gene therapy. Recently, Wang et al. constructed a transgenic PK-15 cell line expressing pCD163 using the PB transposon system, which rendered PK-15 cells susceptible to different PRRSV strains and propagated virus to similar titers compared to the MARC-145 cell line [20]. In the current study, we used the PB system to produce transgenic BHK-21 cells, and subsequently explored whether the PB system represented a feasible and novel approach for achieving multigene co-expression in BHK-21 cells.

In this study, we constructed monoclonal BHK-21 cell lines using the PB transposon system, which expressed pCD163 (porcine CD163) alone, or both pCD163 and pCD169 (porcine CD169), or both pCD163 and sCD151 (simian CD151), or all three molecules: pCD163, pCD169, and sCD151, and distinguished between the contribution of pCD169 and sCD151 to PRRSV infection based on consistent pCD163 expression. We found that the BHK-21 cell line co-expressing all three receptors was more susceptible to PRRSV than MARC-145 cells. Thus, the BHK-21 cell line co-expressing three receptors will be a useful tool for studying receptor-PRRSV interactions, massive PRRSV propagation, and efficient production of the vaccine *in vitro*.

## Materials and Methods

### Cells, plasmids, and viruses

BHK-21 cells and MARC-145 cells were maintained in Dulbecco’s Modified Eagle’s Medium (DMEM) (Gibco, Cat. 11995–073) containing 10% fetal bovine serum (FBS) (Pan biotech, Cat. P30-3302) and 1% penicillin-streptomycin (Gibco, Cat.15140-122). The PB transposon system used in this study contains helper and donor vectors. The following donor vectors were constructed: PB-pCAG-pCD163-ires-Neo, PB-pCAG-pCD169-ires-Puro, and PB-pCAG-sCD151-ires-eGFP. The helper vector expressed the PB transposase (PBase). The BHK-21-STG cell line was generated by electroporation of the pCD163 vector and PBase vector into BHK-21 cells (Table 1). Similarly, BHK-21-DTG1, BHK-21-DTG2, and BHK-21-TTG were generated by electroporation of the corresponding combination of any type of vector; the combination and dosage of plasmid were shown in Table 1. For stable monoclonal cell line selection, transfected cells were cultured in 100 mm dishes by tenfold serial dilutions in the presence of 700 μg/mL G418 (CalBiochem, Cat. 345810). Puromycin (Sigma, Cat. p8833) (2 μg/mL) was used simultaneously for BHK-21-DTG1 and BHK-21-TTG cell selection, as both cell lines were transfected with pCD169 plasmid containing the anti-puromycin gene (Table 1). Antibiotic resistant cell clones were isolated using a cloning cylinder (Sigma, Cat. CLS31668) and transferred into 48-well plates. The selected BHK-21-DTG2 and BHK-21-TTG clones were propagated and further purified by flow cytometry for EGFP expression.

**Table 1:** Transfected plasmid and selection methods of constructed transgenic BHK-21 cells.

Plasmid/Cell lines	BHK-21-STG	BHK-21-DTG1	BHK-21-DTG2	BHK-21-TTG
**PB-pCAG-pCD163-neo**	2.4 μg	1.2 μg	1.2 μg	0.8 μg
**PB-pCAG-pCD169-puro**	-	1.2 μg	-	0.8 μg
**PB-pCAG-sCD151-EGFP**	-	-	1.2 μg	0.8 μg
**PBase-vector**	0.8 μg	0.8 μg	0.8 μg	0.8 μg
**Selection method**	G418	G418+puromycin	G418+FACS	G418+puromycin+FACS
**Monoclone count**	14	13	6	9

“-”: represents plasmid that was not transfected into the corresponding cells.

Two PRRSV strains were used in this study: CH-1a and JXwn06 (both isolated in China). CH-1a is a traditional strain (GenBank accession no. AY032626) and JXwn06 is a highly pathogenic strain (GenBank accession no. EF641008.1). Viruses were propagated in MARC-145 cells.

### RNA isolation, reverse transcription, and qPCR

Total RNA was extracted using the RNeasy Mini Kit (Qiagen, Cat. 74104) following the manufacturer’s instructions. Genomic DNA was removed using DNase I (Qiagen, Cat. 79254). A total of 2 μg of RNA was reverse transcribed for first-strand cDNA synthesis using Moloney murine leukemia virus transcriptase (Promega, Cat. 28025–013) and the oligo (dT) 18 primer. Quantitative RT-PCR (qPCR) analysis was performed using the Roche LightCycler 480 System (LC 480; Roche, Basel, Switzerland) and different primers (Table 2). ORF-7 was used to determine the level of PRRSV mRNA expression. GAPDH from BHK-21 cells (hGAPDH) or MARC-145 cells (sGAPDH) was used as an internal reference gene. To detect IFN-β and IFN-stimulated gene (ISG) expression in infected BHK-21, BHK-21-TTG, and MARC-145 cells, the primers were synthesized and listed in Table 2. Absolute qPCR was used to detect the copy number of PRRSV in the supernatant. Meanwhile, a standard set of mixtures (representing 1, 2, 4, 8, 16, and 32 copies of plasmid DNA consisting of ORF7 sequence) was used to generate a standard curve to determine the correlation between the Ct value and the copy number of virions.

**Table 2:** Quantitative RT-PCR primers list.

Primer	Sequence (5’→3’)
p-cd163-F	CCACAGGTCGCTCATCTTTT
p-cd163-R	CTGCCTCTGTCTTCGCTTCT
p-cd169-F	GGGTGTGGAGATCCTTTTCA
p-cd169-R	CCTGAGGGTTGCTGCTATTC
s-cd151-F	ACCGTTTGCCTCAAGTACCT
s-cd151-R	AGATGCCCACTGCCATGACA
ORF-7-F	AATAACAACGGCAAGCAGCA
ORF-7-R	GCACAGTATGATGCGTCGGC
h-gapdh-F	GACTTCAACAGTGACTCCCAC
h-gapdh-R	TCTGTTGCTGTAGCCAAATTC
h-ifn-b2-F	CTCCGCAAGAGACTTCCATC
h-ifn-b2-R	ACCAAACCTCCGACTTGTT
h-ccl2-F	TTCTGTGCCTACTGCTCACG
h-ccl2-R	GGGATCTTTCTGGCATTGAA
h-rnasel-F	CCAGAGGGTAAAAACGTGGA
h-rnasel-R	TGCACCAAACCTGTGTGTTT
h-mx1-F	CTTCAAGGAGCACCCACACT
h-mx1-R	CTTGCCCTCTGGTGACTCTC
s-gapdh-F	ACCCAGAAGACTGTGGATGGTCGCTGTTGAAGTCAGAGGA
s-gapdh-R	TCGCTGTTGAAGTCGGAGGAACCCAGAAGACTGTGGATGG
s-ifn-b2-F	TACATCCTCGACGGCATCTC
s-ifn-b2-R	AGTGCCTCTTTGCTGCTTTC
s-ccl2-F	GCAGCAAGTGTCCCAAAGAA
s-ccl2-R	TGGGTTGTGGAGTGAGTGTTC
s-rnasel-F	TCCTGGCAGTGGAGAAGAAG
s-rnasel-R	TGTTTTGCCGTCACTGTCTG
s-mx1-F	CGGCTTGCTTTCACAGATGT
s-mx1-R	GCTTCTCGCCTTCTCTCTCTT

The first letter of each primer represents different specie: “p”, porcine; “s”, simian; “h”, hamster.

### Immunofluorescence assay (IFA)

Cells were fixed with cold methanol-acetone (1:1) for 15 min at 4°C and washed with phosphate-buffered saline (PBS) three times. After blocking with 1% BSA–PBS for 30 min at room temperature, the cells were incubated with antibodies against pCD163 (mAb 2A10, AbD Serotec, and rabbit anti- pCD163 polyclonal antibody, produced by Genscript Company, CHINA), or pCD169 (rabbit anti-pCD169 polyclonal antibody, produced by Genscript Company, CHINA) or sCD151 (mAb 11G5a, AbD Serotec) at 37°C for 1 h. After washing with PBS three times, the cells were incubated with an Alexa Fluor 488-labeled goat anti-rabbit IgG antibody (Invitrogen, Cat. A-11008) or Alexa Fluor 594-labeled goat anti-mouse IgG antibody (Invitrogen, Cat. A-11005) at 37°C for 1 h. Finally, cells were counterstained with DAPI and examined using a fluorescence microscope. For detection of PRRSV infection in transgenic BHK-21 cells and MARC-145 cells, the PRRSV N protein was stained with SDOW17 (mAb; Rural Technologies, Cat. HB-10997) and observed using Alexa Fluor 594-labeled goat anti-mouse IgG antibody staining.

### Viral infection and titration

Cells were incubated with JXwn06 or CH-1a viruses for 2 hours, and then replaced with virus-free medium after washing with PBS. The cells or supernatants were collected for virus RNA and protein detection at the indicated time points. To determine viral titers, the infected BHK-21-TTG and MARC-145 cells, together with supernatants, were frozen and thawed three times. MARC-145 cells were seeded in a 96-well plate and infected with serially diluted cell lysates. Cells were stained for PRRSV N protein at 72 hpi. The viral titer was calculated using the Reed-Muench method and expressed as 50% tissue culture infective doses (TCID50) per milliliter.

## Results

### Generation and identification of BHK-21 cells expressing PRRSV receptor(s)

**Fig 1:**
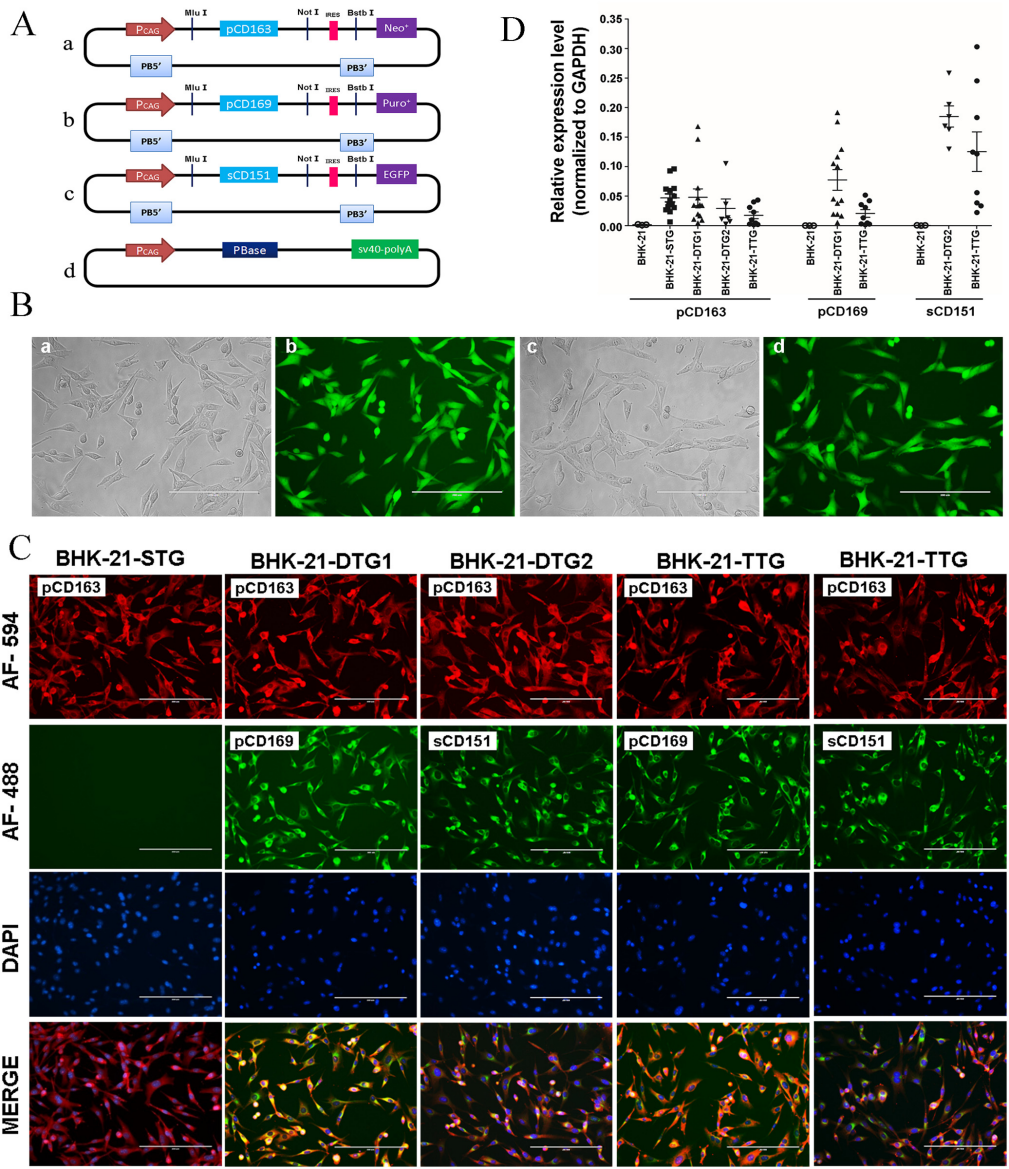
Generation of PRRSV receptor transgenic BHK-21 cells. (A) Schematic representation of PRRSV receptor constructs for transfection. (a–c) Donor plasmids including the PiggyBac 3’ terminator, CAG promoter, IRES sequence, and selection marker are indicated with bars. The pCD163 cDNA (a), pCD169 cDNA (b), and sCD151 cDNA (c) are indicated. MluI, NotI, and BstbI restriction sites are shown. (d) Helper vector structure expressing the PBase. (B) Detection of EGFP fluorescence in the BHK-21-DTG2 and BHK-21-TTG cell lines. The selected BHK-21-DTG2 and BHK-21-TTG monoclones were imaged using a fluorescence microscope under white light field (panels a and c) and the UV light field (panels b and d) (Bar = 200 μm). (C) pCD163 was surface stained with a mouse anti-pig monoclonal antibody coupled by Alexa 594-conjugated goat anti-mouse secondary antibody in BHK-21-STG, BHK-21-DTG1; and BHK-21-TTG cells, or a rabbit anti-pig polyclonal antibody coupled by 594-conjugated goat anti-rabbit secondary antibody in BHK-21-DTG2 and BHK-21-TTG cells correspondingly; pCD169 was surface stained with a rabbit anti-pig polyclonal antibody coupled by Alexa 488-conjugated goat anti-rabbit secondary antibody; sCD151 was surface stained with a mouse anti-human monoclonal antibody coupled by Alexa 488-conjugated goat anti-mouse secondary antibody. Bar = 200 μm. (D) The mRNA expressions of pCD163, pCD169, and sCD151 in transgenic BHK-21 monoclones were analyzed by qPCR and the target gene expression was calculated and normalized to GAPDH based on the 2^-Δct^ method.

**Fig 2:**
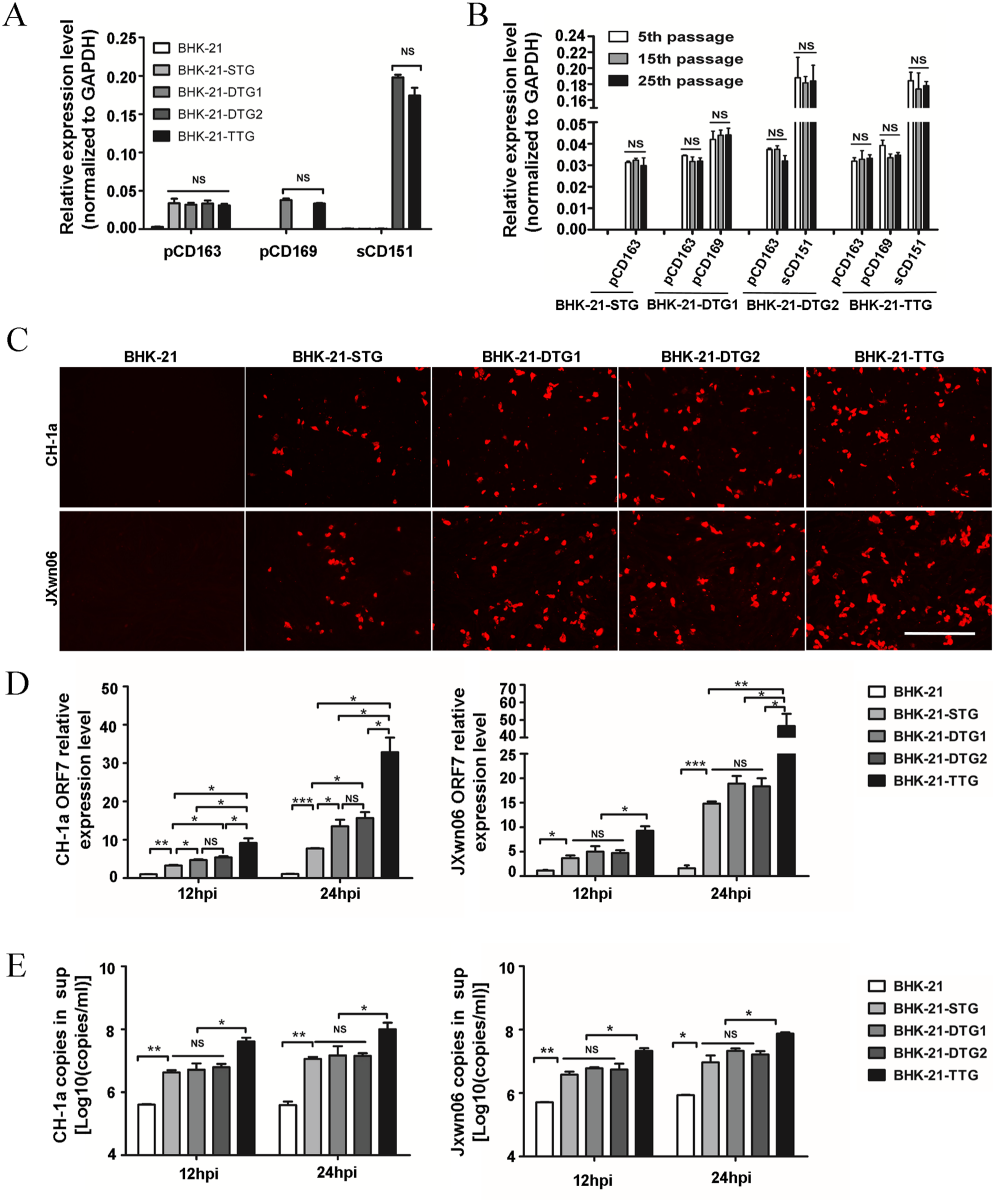
Susceptibility analysis of PRRSV receptor transgenic cell lines. (A) Quantitative RT-PCR (qPCR) analysis of pCD163, pCD169, and sCD151 mRNA expression in transgenic BHK-21 cells: the respective expressions of the target genes were calculated and normalized to GAPDH using the 2^-Δct^ method. (B) qPCR analysis of pCD163, pCD169, and sCD151 mRNA expression in transgenic BHK-21 cells at the 5^th^,15^th^, and 25^th^ passage. Cells were collected and total RNA was extracted, reverse transcribed, and quantitated by qPCR. (C) Immunofluorescence assay (IFA) analysis of PRRSV N protein expression in transgenic BHK-21 cells infected with PRRSV. Transgenic cells were infected with PRRSV CH-1a or JXwn06 (MOI = 1), and expression of the N protein was examined at 36 hpi using the monoclonal antibody SDOW17 and a secondary antibody conjugated to Alexa Fluor 594. Bar = 200 μm. (D) qPCR analysis of viral ORF7 RNA expression in BHK-21 transgenic cells at 12 hpi and 24 hpi. The four transgenic BHK-21 cells were infected with PRRSV CH-1a or JXwn06 (MOI = 0.5) and viral ORF7 in the cells was analyzed by qPCR at 12 hpi and 24 hpi. (E) The supernatant containing PRRSV RNA was analyzed based on absolute quantitative RT-PCR at the indicated time points. Transgenic cells were infected with PRRSV CH-1a or JXwn06 (MOI = 0.5), and the supernatant was collected and used for RNA extraction and absolute qPCR of the virions at 12 hpi and 24 hpi. The data were representative of the results of three independent experiments (mean ± SD). Statistical significances were analyzed using Student’s t-test. *, P<0.05; **, P<0.01; ***, P<0.001; NS, not significant.

To obtain transgenic cell lines expressing PRRSV receptor(s), we co-transfected one or multiple donor vectors (Fig 1A), together with the helper vector, into BHK-21 cells (Table 1). A total of 14 BHK-21-STG clones, 13 BHK-21-DTG1 clones, 6 BHK-21-DTG2 clones, and 9 BHK-21-TTG clones were selected, as shown in Table 1. Furthermore, BHK-21-DTG2 and BHK-21-TTG clones were sorted by flow cytometry for EGFP and the purity was above 95% (Fig 1B). EGFP was connected with sCD151 cDNA based on the IRES sequence, and was transcribed along with the sCD151 gene under control of the CAG promoter (Fig 1A and 1Aa–1Ac), after which sCD151 expression could be assumed [41]. To evaluate the influence of the endogenous genes, the expression level of CD163, CD151, and CD169 of BHK-21 cell were detected. Both CD163 and CD151 were at very low expression levels, and CD169 was too low to be detected (S1 Fig). The presence of exogenous PRRSV receptor expression was confirmed based on immunofluorescent staining. In all transgenic clones, cells could stably express exogenous genes (Fig 1C). Since CD163 is considered the most essential factor for PRRSV entry, it is necessary and sufficient to render insensitive cell lines fully permissive to PRRSV [17,25]. Therefore, each transgenic cell clone we constructed expressed pCD163, and the mRNA quantities in all transgenic cell lines were evaluated (Fig 1D). To eliminate the influence of pCD163 copy numbers, clones with similar expression levels of pCD163 were selected to analyze the function of pCD169 and sCD151 in transgenic cell lines. The selection was limited by the expression level of pCD163 in BHK-21-DTG2 and BHK-21-TTG cell lines (Fig 1D). Finally, four transgenic clones with similar expression levels of corresponding transgenes were selected (Fig 2A) to perform the following experiments.

### PRRSV infection and replication in transgenic cells with different receptors

To determine whether pCD169 and sCD151 could synergistically enhance PRRSV infection in pCD163 transgenic cells, PRRSV infection assays were performed. pCD163, pCD169, and sCD151 expression of selected clones were further confirmed prior to PRRSV infection. As shown in Fig 2A, the receptor expression quantity of the four clones was stable and consistent after several passages. To validate the integrated stability of exogenous genes during cell passages, the receptor expression levels of selected clones were verified by qPCR at the 5^th^, 15^th^, and 25^th^ passage. It was clear that all clones could stably express the transgenic exogenous genes after at least 25 passsages, and no clones lost any gene expression (Fig 2B). To compare the growth speed and viability, the same initial cells (5×10^4^) of all cell lines were seeded to culture and checked at designed time points. All transgenic clones had the similar cell viability and doubling times with the parental BHK-21 cells, which showed advantages comparing with MARC-145 cells (S2 Fig). Transgenic cells and BHK-21 cells were challenged with JXwn06 or CH-1a at an MOI of 1. For intracellular viral replication, the PRRSV N protein was analyzed by IFA at 36 hpi. Compared with the abortive infection in BHK-21 cells, all transgenic cells supported viral infection (Fig 2C). However, the amount of virions differed among the four transgenic clones. Compared with other transgenic cells, BHK-21-TTG cells showed the most intensely red fluorescence of N protein after challenge by two PRRSV strains. BHK-21-DTG1 and BHK-21-DTG2 cells showed more fluorescence than BHK-21–STG cells. To further compare the efficiency of infection among all transgenic cell lines, the cells were incubated with JXwn06 or CH-1a and mRNA levels of ORF7 in cells and supernatants were analyzed by qPCR. As shown in Fig 2D, JXwn06 or CH-1a viral loads in BHK-21-TTG were highest compared to other transgenic cells after challenge by two PRRSV strains. The viral loads in BHK-21-DTG1 and DTG2 were significantly higher than in BHK-21-STG cells after CH-1a challenge, but there were no differences in viral loads in BHK-21-DTG1 and DTG2 compared with BHK-21-STG cells after JXwn06 challenge. Consistent with intracellular virus replication, BHK-21-TTG cells could release most virions to the supernatant (Fig 2E), but there was no difference in viral loads in supernatants of BHK-21-DTG1 and DTG2 compared with BHK-21-STG cells after challenge by the two strains. Taken together, our results demonstrated that all transgenic cells were susceptible to PRRSV infection, and the BHK-21-TTG cells could be achieved the most efficient results.

### Comparison of PRRSV infectivity in BHK-21-TTG and MARC-145 cells

**Fig 3:**
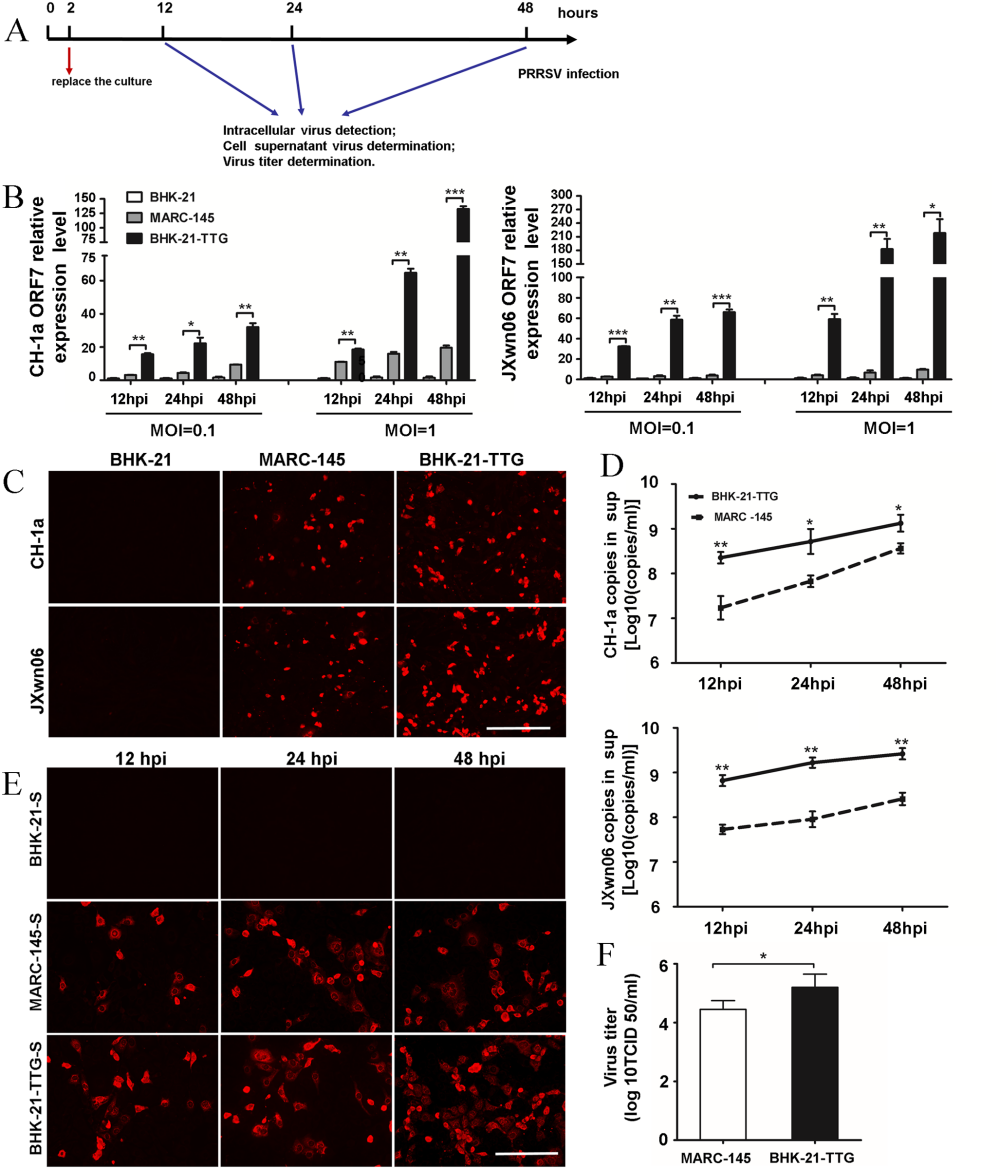
Comparison of PRRSV infection efficiency between BHK-21-TTG and MARC-145 cells. (A) Schematic of the experimental design for the detection of PRRSV infection in different transgenic BHK-21 cells at the indicated time points. The cells were incubated with CH-1a or JXwn06 (MOI = 1) for 2 h, and medium containing virus was replaced with fresh medium at 2 hpi after washing the cells. The viruses were then detected using the indicated methods. (B) Viral ORF7 RNA of the two PRRSV strains, CH-1a and JXwn06, was determined at the indicated times by qPCR. Three wells of each cell line at each time point were used. Data were presented as means ± SD of at least three independent experiments. (C) Representative IFA of the PRRSV N protein at 36 hpi. BHK-21, BHK-21-TTG, and MARC-145 cells were inoculated with JXwno6 or CH-1a (MOI = 1). PRRSV was detected using the monoclonal anti-N protein antibody (SDOW17) followed by a secondary antibody conjugated to Alexa Fluor 594. BHK-21 cells were used as the negative control. (Bar = 200 μm). (D) Supernatant containing PRRSV RNA was analyzed by absolute quantitative RT-PCR at the indicated time points. (E) IFA of the PRRSV N protein in MARC-145 cells after being inoculated with supernatant from BHK-21, MARC-145, and BHK-21-TTG cells. The supernatant (BHK-21-S, MARC-145-S, and BHK-21-TTG-S) of infected cells was collected at 36 hpi, and inoculated into MARC-145 cells by 100 μL/well plus 100 μL medium in 48 well plates, after which IFAs for N protein in MARC-145 were performed at 36 hpi (Bar = 200 μm). (F) Titer determination of the PRRSV strains in MARC-145 and BHK-21-TTG cells. MARC-145 and BHK-21-TTG cells were infected with JXwn06 PRRSV at an MOI of 1. At 48 hpi, viruses were extracted by freezing and thawing three times and the virus titers (TCID_50_/ml) in MARC-145 cells were measured. Each data point represents the mean ± SD from three independent experiments. Statistical significances were analyzed by Student’s t-test. *, P<0.05; **, P<0.01; ***, P<0.001.

MARC-145 cells can be infected with PRRSV particularly due to the expression of endogenous CD163 and CD151, and have already been applied for PRRSV studies as a common cell platform. Therefore, we compared BHK-21-TTG cells and MARC-145 cells using different methods (Fig 3A) to verify the susceptibility of the new cell line. First, quantitative PCR results demonstrated the expression levels of both endogenous CD163 and CD151 in MARC-145 cells were significantly lower than the transgenes in BHK-21-TTG cells, whereas the CD169 of MARC-145 were too low to be detected (S1 Fig). Subsequently, BHK-21-TTG and MARC-145 cells were infected with CH-1a and JXwn06 at the three indicated dosages. Viral RNA accumulation was significantly higher in BHK-21-TTG cells than in MARC-145 cells under the two dosages (MOI = 0.1 or 1) at different time points (12 hpi, 24 hpi, 48 hpi), as determined by qPCR (Fig 3B). This result was further confirmed based on intracellular PRRSV N protein staining at 36 hpi, indicating that BHK-21-TTG cells were more susceptible (Fig 3C). To further explore whether the virus could be released from the cytoplasm into the supernatant, PPRSV levels in the cell supernatants at different time points were measured using qPCR and IFA. The results showed that the infected BHK-21-TTG cells released more progeny virions than MARC-145 cells (Fig 3D and 3E). Moreover, viral titers produced from the two cell lines were measured (Fig 3F). This result demonstrated that the cell lysates from infected BHK-21-TTG cells (TCID50 = 5.20) had stronger infectivity than those from MARC-145 cells (TCID50 = 4.45). These results showed that BHK-21-TTG cells fully supported PRRSV infection and reproductive replication, and also sustained complete life cycles. In summary, the three-receptor integrated BHK-21 cells showed more robust infectivity to PRRSV than MARC-145 cells.

### ISG responses to PRRSV were down-regulated in BHK-21-TTG cells

**Fig 4:**
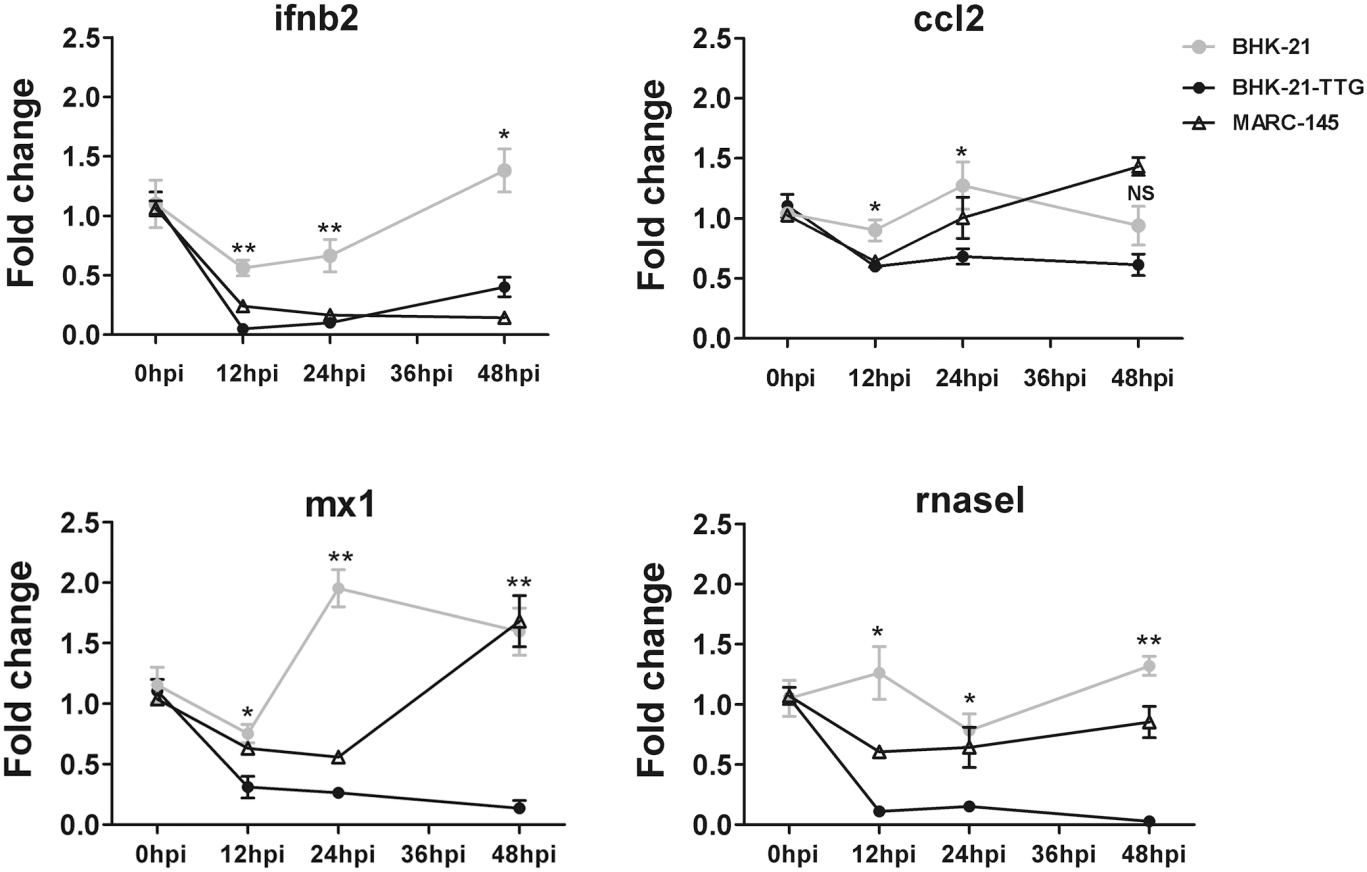
IFN and ISG responses to PPRSV were suppressed in BHK-21-TTG cells. BHK-21, BHK-21-TTG, and MARC-145 cells were infected with JXwn06 at an MOI of 1, and the expression of intracellular ISGs was examined by qPCR at the indicated time points. The fold change was calculated using the 2^−ΔΔCt^ method. ΔCt(hpi) = Ct (target gene) –Ct (GAPDH); ΔCt (reference) = Ct (0 hpi target gene)–Ct (GAPDH); ΔΔCt = ΔCt (hpi) −ΔCt (reference). hpi represented genes at the corresponding time point; 0 hpi represented gene expression before infection, with a default fold of gene RNA at 0 hpi of 1. Three wells of each cell type at each time point were used. Data were presented as means ± SD of at least three independent experiments. Statistical significances were analyzed by Student’s t-test. *, P<0.05; **, P<0.01; NS, not significant.

Type I interferon orchestrates a protective response by inducing ISG production at early time points after virus infection [42,43]. PRRSV is known to inhibit the type I IFN response following ISG production through different mechanisms [44,45]. PRRSV interferes with the RIG-I dependent pathway to inhibit type I IFN production; for example, Nsp1 could inhibit activation of the NF-κB-responsive promoter and inhibit IRF3 nuclear translocation, both of which result in the reduction of IFN [46,47]. On the other hand, PRRSV could inhibit IFN-mediated JAK-STAT signaling pathways to interrupt ISG transcription; for example, *ISG15*, *ISG56*, *CCL2*, *MX1*, *OAS2*, and *RNaseL* of PAMs were significantly downregulated after infection with the PRRSV strain VR2385 [48]. To analyze the IFN response to PPRSV, BHK-21-TTG, BHK-21, and MARC-145 cells were infected with JXwn06. IFN and ISG mRNA expression levels were determined by qPCR after infection. IFN-β expression and several ISGs, including *CCL2*, *MXI*, and *RNaseL*, were lower at 12 hpi, 24 hpi, and 48 hpi in BHK-21-TTG cells compared with BHK-21 cells. *IFN-β* (ifnb2) mRNA expression was suppressed by 5.8-fold at 12 hpi, 6.6-fold at 24 hpi, and 7.7-fold at 48 hpi in BHK-21-TTG cells compared with BHK-21 cells. *MX1* mRNA levels were similarly decreased in BHK-21-TTG compared with BHK-21 cells. *CCL2* and *RNase L* were inhibited by JXwn06 infection compared with BHK-21 cells (Fig 4). IFN and ISGs of MARC-145 cells were also decreased at 12 hpi and 24 hpi compared to 0 hpi, and the degree of reduction was modest than in BHK-21-TTG cells. At 48 hpi, three ISGs (*MX1*, *CCL2*, and *RNase L*) showed recovery though IFN-β sequentially decreased in MARC-145 cells.

## Discussion

PRRSV, which infects pigs, has caused devastating economic losses worldwide; however, the pathogenesis and vaccine development of PRRSV have been limited due to a lack of efficient cell lines and small animal models. The complete life cycle of PRRSV infection comprises multiple steps, including recognition, attachment, internalization, uncoating, genome release, and assembly. Several molecules involved in the process of PRRSV infection have been identified on PAMs or MARC-145 cells. Some studies have shown that CD163 alone mediates PRRSV entry and the production of progeny viruses [17, 20]. CD169, a known receptor of attachment and internalization for PRRSV infection, cannot promote viral uncoating and genome release in PRRSV non-permissive cells [14,15]. However, CD169 transfection enhances the infection efficiency of non-permissive cell lines expressing CD163 [18,26]. CD151, another PRRSV receptor identified on MARC-145 cells, interacts with the 3’ UTR of PRRSV RNA. At this time, studies on the synergic interaction of these three receptors have not been reported.

The PB transposon system is commonly used to construct transgenic cells and animals due to its high integration efficiency. In the present study, we developed BHK-21 derived cell lines transfected with different PRRSV receptors using the PB transposon system. Since pCD163 is the most essential factor for the PRRSV infection cycle, all cell lines were transfected by pCD163 with/without pCD169 and sCD151 plasmids. Four types of transgenic BHK-21 cells were obtained, including BHK-21-STG (only expressing pCD163), BHK-21-DTG1 (expressing both pCD163 and pCD169), BHK-21-DTG2 (expressing both pCD163 and sCD151), and BHK-21-TTG (expressing pCD163, pCD169, and sCD151 together). After more than 25 passages, transgenic cells showed similar cell viability and doubling times compared to their parental BHK-21 cells, and every clone stably expressed exogenous genes. To explore the effects of pCD169 and sCD151 based on the same level of pCD163, mRNA levels of receptors in transgenic cell lines were quantitated, and clones with similar expression level of each receptor were used to perform the following experiments. Viral infection results demonstrated that, under similar pCD163 expression conditions, either pCD169 or sCD151 transgenic cells showed slightly increased virion loads compared to pCD163-alone transgenic cells. By contrast, cells that co-expressed the three receptors were significantly enhanced to PRRSV JXwn06 or CH-1a, which indicated that the cooperation of pCD163, pCD169, and sCD151 was important to enhance the infection efficiency of PRRSV. Importantly, the results of a highly pathogenic strain, JXwn06, were consistent with that of a traditional strain, CH-1a, indicating that viral infection of different transgenic cells was not strain-specific.

Currently, vaccination is the primary approach for PRRSV prevention and control. PRRSV infects and replicates efficiently in cultivated PAMs *ex vitro*. However, due to batch variation, risk of pathogen contamination, and high economic cost [26], this type of cell cannot be used for vaccine production. PRRSV-susceptible cell lines, such as MARC-145, have overcome the problems associated with the primary macrophages. Nevertheless, only some PRRSV strains could efficiently infect MARC-145 cells [49,50]; thus, adaptation of the virus for growth on monkey-derived cells is required, due to a different entry pathway compared with porcine macrophages [30,31]. To obtain better cell lines than MARC-145, a new cell line stably expressing more functional receptors to simulate the natural course of PRRSV infection is required. In this study, we obtained BHK-21-TTG cells co-expressing CD163, CD169, and CD151, in which the viral loads of intracellular, supernatant or both significantly exceeded that in MARC-145 cells. Comparing to the endogenous receptors of MARC145 cells, BHK-21-TTG cells have higher expression levels of transgenes, and could be stably serially passaged without any decreased infectivity. In general, several advantages on basic profiles were also displayed in BHK-21-TTG cells, including cell viability, doubling times, and growth speed.

Type I IFNs could trigger activation of the JAK/STAT signaling pathway and induce expression of a wide variety of ISGs that can establish antiviral and immune-regulatory states [44,45]. Previous studies reported that PRRSV escapes host antiviral immune responses by strongly inhibiting downstream signal transduction of type I IFNs and subsequently interdicting ISG transcription [51,52]. Our results demonstrated that the expressions of *IFNβ*, *CCL2*, *MX1*, and *RNaseL* were inhibited in BHK-21-TTG cells at least within 48 hpi, while MARC-145 cells were inhibited only until 24 hpi. This indicated that the BHK-21-TTG cell line could also trigger a longer type I IFN response induced by PRRSV infection, which is a useful feature of the BHK-21-TTG cell line that allows it to imitate natural host cells *in vitro*.

In summary, a BHK-21 derived cell line co-expressing three receptors (pCD163, pCD169, and sCD151) was constructed, and showed increased susceptibility to PRRSV compared to MARC-145, thereby supported the effectiveness of receptor synergistic action in transgenic cell lines on viral infection. In this study, we provide an alternative approach for vaccine production and virus isolation instead of MARC-145 cells. The new transgenic cell line was very stable during long-term serial passages and could reflect the type I IFN response, similar to PRRSV induction in PAMs and MARC-145 cells. Thus, our study provides two important advances: first, the triple transgenic BHK-21 cells can substitute MARC-145 as a useful tool for various *in vitro* studies of PRRSV with respect to host cell interactions, viral pathogenesis, and the mechanism of immunity. In addition, our results provide useful experimental data for developing a rodent model for PRRSV studies using a similar approach.

## Supporting Information

S1 Fig Analysis of CD163, CD169, and CD151 mRNA expression in BHK-21, BHK-21-TTG and MARC-145 cells by quantitative PCR. The endogenous CD163, CD169, and CD151 in both BHK-21 and MARC-145 cells as well as the corresponding transgenic receptors of BHK-21-TTG were detected. The relative expression levels were normalized to endogenous GAPDH. The data were representative from three independent experiments with similar results (mean ± SD). Statistical significance was analyzed by Student’s t-test. *, P<0.05; **, P<0.01; ***, P<0.001. The primers of endogenous genes for the BHK-21 and MARC-145 cells were listed as follows: BHK-21 primers (hamster): hCD163-F: 5’- CTCAGGAAACCAATCCCAGA-3’; hCD163-R: 5’-GCCTCCATTTACCAAACGAA-3’; hCD169-F: 5’-CCTACAACTTCCGCTTCGAG-3’; hCD169-R: 5’-CTGGGGTCCT TTGTCACAGT-3’; hCD151-F: 5’-GCTGTGCCAC TTTCAAGGAG-3’; hCD151-R: 5’-GCATTCGTCA CACCATCTTG-3’; hGAPDH-F: 5’-GACTTCAACAGTGACTCCCAC-3’; hGAPDH-R: 5’-TCTGTTGCTGTAGCCAAATTC-3’; MARC-145 primers (simian): sCD163-F: 5’-ACTGCTCTGGGTGCTTCACT-3’; sCD163-R: 5’-CGACCTCCTC CATTTACCAA-3’; sCD169-F: 5’-CCTTCACTGCTCTGTGGTCA-3’; sCD169-R: 5’-TGTCAGCTTC CTCCAGGTCT-3’; sCD151-F: 5’-ACCGTTTGCCTCAAGTACCT-3’; sCD151-R: 5’-AGATGCCCACTGCCATGACA-3’; sGAPDH-F: 5’- ACCCAGAAGACTGTGGATGG -3’; sGAPDH-R: 5’- TCGCTGTTGAAGTCGGAGGA -3’.
(TIF)

S2 Fig Cell culture and cellularity of BHK-21, MARC-145 and BHK-21 transgenic cells. Initially, 5×10^4^ cells of BHK-21, BHK-21-STG, BHK-21-DTG1, BHK-21-DTG2, BHK-21-TTG and MARC-145 cells were seeded in 24 well plates and counted after 24 h, 36 h, 48 h and 60 h. The data were representative from three independent experiments (mean ± SD). Statistical significance was analyzed by Student’s t-test. There was significant difference in growth speed and doubling times between BHK-21-TTG and MARC-145 cells (p value was 0.0215, 0.0449, 0.0008, 0.0004 at 24 h, 36 h, 48 h and 60 h, respectively), but no significant difference in growth speed and viability of transgenic cells comparing with parental BHK-21 cells.
(TIF)
