# Correction: Developing a Triple Transgenic Cell Line for High-Efficiency Porcine Reproductive and Respiratory Syndrome Virus Infection

**DOI:** 10.1371/journal.pone.0160325

**Published:** 2016-07-26

**Authors:** Linlin Zhang, Zhengzhi Cui, Lei Zhou, Youmin Kang, Li Li, Jinxiu Li, Yunping Dai, Shuyang Yu, Ning Li

There are errors in Table 2. The sequences for s-gapdh-F and s-gapdh-R appear incorrectly in the published article. Please see the correct Table 2 and its caption here.

**Table 2:** Quantitative RT-PCR primers list.

Primer	Sequence (5’→3’)
p-cd163-F	CCACAGGTCGCTCATCTTTT
p-cd163-R	CTGCCTCTGTCTTCGCTTCT
p-cd169-F	GGGTGTGGAGATCCTTTTCA
p-cd169-R	CCTGAGGGTTGCTGCTATTC
s-cd151-F	ACCGTTTGCCTCAAGTACCT
s-cd151-R	AGATGCCCACTGCCATGACA
ORF-7-F	AATAACAACGGCAAGCAGCA
ORF-7-R	GCACAGTATGATGCGTCGGC
h-gapdh-F	GACTTCAACAGTGACTCCCAC
h-gapdh-R	TCTGTTGCTGTAGCCAAATTC
h-ifn-b2-F	CTCCGCAAGAGACTTCCATC
h-ifn-b2-R	ACCAAACCTCCGACTTGTT
h-ccl2-F	TTCTGTGCCTACTGCTCACG
h-ccl2-R	GGGATCTTTCTGGCATTGAA
h-rnasel-F	CCAGAGGGTAAAAACGTGGA
h-rnasel-R	TGCACCAAACCTGTGTGTTT
h-mx1-F	CTTCAAGGAGCACCCACACT
h-mx1-R	CTTGCCCTCTGGTGACTCTC
s-gapdh-F	ACCCAGAAGACTGTGGATGG
s-gapdh-R	TCGCTGTTGAAGTCGGAGGA
s-ifn-b2-F	TACATCCTCGACGGCATCTC
s-ifn-b2-R	AGTGCCTCTTTGCTGCTTTC
s-ccl2-F	GCAGCAAGTGTCCCAAAGAA
s-ccl2-R	TGGGTTGTGGAGTGAGTGTTC
s-rnasel-F	TCCTGGCAGTGGAGAAGAAG
s-rnasel-R	TGTTTTGCCGTCACTGTCTG
s-mx1-F	CGGCTTGCTTTCACAGATGT
s-mx1-R	GCTTCTCGCCTTCTCTCTCTT

The first letter of each primer represents different specie: “p”, porcine; “s”, simian; “h”, hamster.
