# Supplementary figures and images for: Developing a Triple Transgenic Cell Line for High-Efficiency Porcine Reproductive and Respiratory Syndrome Virus Infection
Source: PLoS One. 2016 May 16;11(5):e0154238. doi: 10.1371/journal.pone.0154238 (PMC4868347; doi:10.1371/journal.pone.0154238)

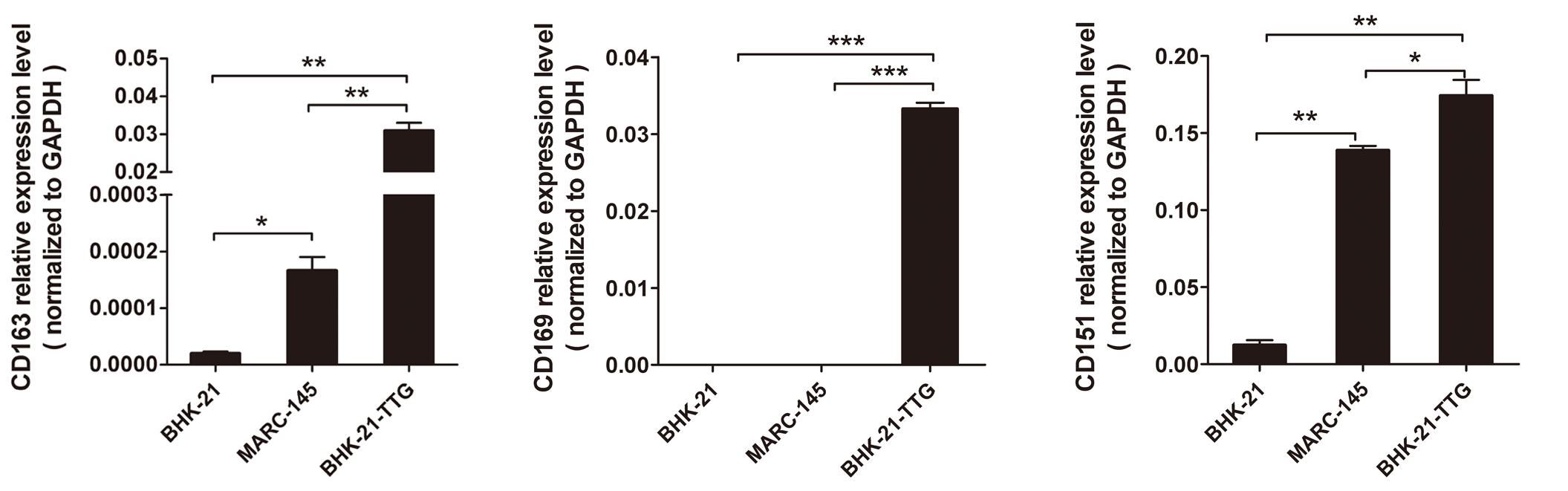

Supplement: S1 Fig — The endogenous CD163, CD169, and CD151 in both BHK-21 and MARC-145 cells as well as the corresponding transgenic receptors of BHK-21-TTG were detected. The relative expression levels were normalized to endogenous GAPDH. The data were representative from three independent experiments with similar results (mean ± SD). Statistical significance was analyzed by Student’s t-test. *, P<0.05; **, P<0.01; ***, P<0.001. The primers of endogenous genes for the BHK-21 and MARC-145 cells were listed as follows: BHK-21 primers (hamster): hCD163-F: 5’- CTCAGGAAACCAATCCCAGA-3’; hCD163-R: 5’-GCCTCCATTTACCAAACGAA-3’; hCD169-F: 5’-CCTACAACTTCCGCTTCGAG-3’; hCD169-R: 5’-CTGGGGTCCT TTGTCACAGT-3’; hCD151-F: 5’-GCTGTGCCAC TTTCAAGGAG-3’; hCD151-R: 5’-GCATTCGTCA CACCATCTTG-3’; hGAPDH-F: 5’-GACTTCAACAGTGACTCCCAC-3’; hGAPDH-R: 5’-TCTGTTGCTGTAGCCAAATTC-3’; MARC-145 primers (simian): sCD163-F: 5’-ACTGCTCTGGGTGCTTCACT-3’; sCD163-R: 5’-CGACCTCCTC CATTTACCAA-3’; sCD169-F: 5’-CCTTCACTGCTCTGTGGTCA-3’; sCD169-R: 5’-TGTCAGCTTC CTCCAGGTCT-3’; sCD151-F: 5’-ACCGTTTGCCTCAAGTACCT-3’; sCD151-R: 5’-AGATGCCCACTGCCATGACA-3’; sGAPDH-F: 5’- ACCCAGAAGACTGTGGATGG -3’; sGAPDH-R: 5’- TCGCTGTTGAAGTCGGAGGA -3’. (TIF) [file pone.0154238.s001.tif]

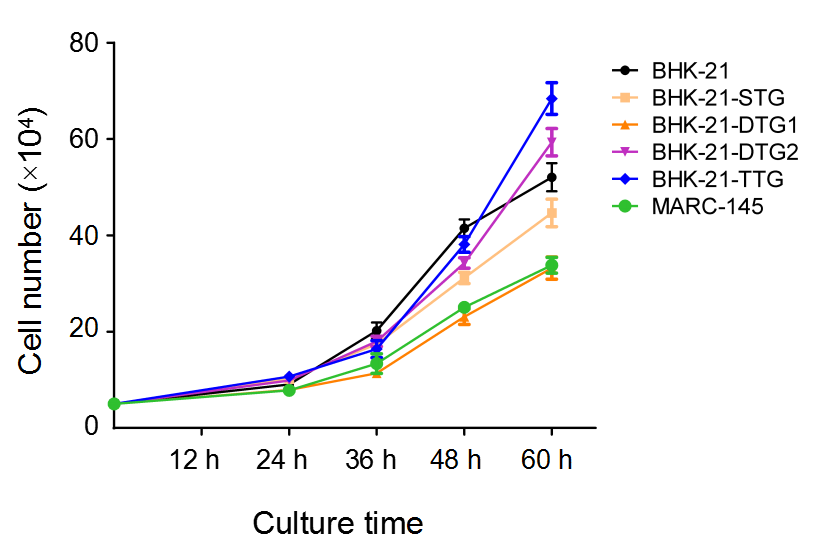

Supplement: S2 Fig — Initially, 5×10^4^ cells of BHK-21, BHK-21-STG, BHK-21-DTG1, BHK-21-DTG2, BHK-21-TTG and MARC-145 cells were seeded in 24 well plates and counted after 24 h, 36 h, 48 h and 60 h. The data were representative from three independent experiments (mean ± SD). Statistical significance was analyzed by Student’s t-test. There was significant difference in growth speed and doubling times between BHK-21-TTG and MARC-145 cells (p value was 0.0215, 0.0449, 0.0008, 0.0004 at 24 h, 36 h, 48 h and 60 h, respectively), but no significant difference in growth speed and viability of transgenic cells comparing with parental BHK-21 cells. (TIF) [file pone.0154238.s002.tif]
